# Crystal structure of *N*,*N*,*N*-tris­[(1,3-benzo­thia­zol-2-yl)meth­yl]amine

**DOI:** 10.1107/S2056989015017417

**Published:** 2015-09-26

**Authors:** Velabo Mdluli, James A. Golen, Arnold L. Rheingold, David R. Manke

**Affiliations:** a Department of Chemistry and Biochemistry, University of Massachusetts Dartmouth, 285 Old Westport Road, North Dartmouth, MA 02747, USA; b Department of Chemistry, University of California, San Diego, 9500 Gilman Drive, La Jolla, CA 92093, USA

**Keywords:** crystal structure, benzo­thia­zoles, C—H⋯N inter­actions

## Abstract

The title compound, C_24_H_18_N_4_S_3_, exhibits three near planar benzo­thia­zole systems in a pseudo-*C*_3_ conformation. The dihedral angles between the planes of the benzo­thia­zole groups range from 112.56 (4) to 124.68 (4)° In the crystal, mol­ecules are connected to each other through three short C—H⋯N contacts, forming an infinite chain along [100]. The molecules are also linked by π–π interactions with each of the three five-membered thiazole rings. [inter-centroid distance range: 3.614 (1)–4.074 (1) Å, inter-planar distance range: 3.4806 (17)–3.6902 (15) Å, slippage range: 0.759 (3)–1.887 (3) Å].

## Related literature   

For synthesis of the title compound and a structure of the ligand bound to copper, see: Thompson *et al.* (1980 ▸). For a related organic structure, see: Zhang *et al.* (2009 ▸). For other related structures, see; Bautista & Thompson (1980 ▸); Pandey & Mathur (1995 ▸). For a study of its use as a ligand in azide–alkyne cyclo­additions, see: Rodionov, Presolski, Gardinier *et al.* (2007 ▸); Rodionov, Presolski, Diaz *et al.* (2007 ▸). 
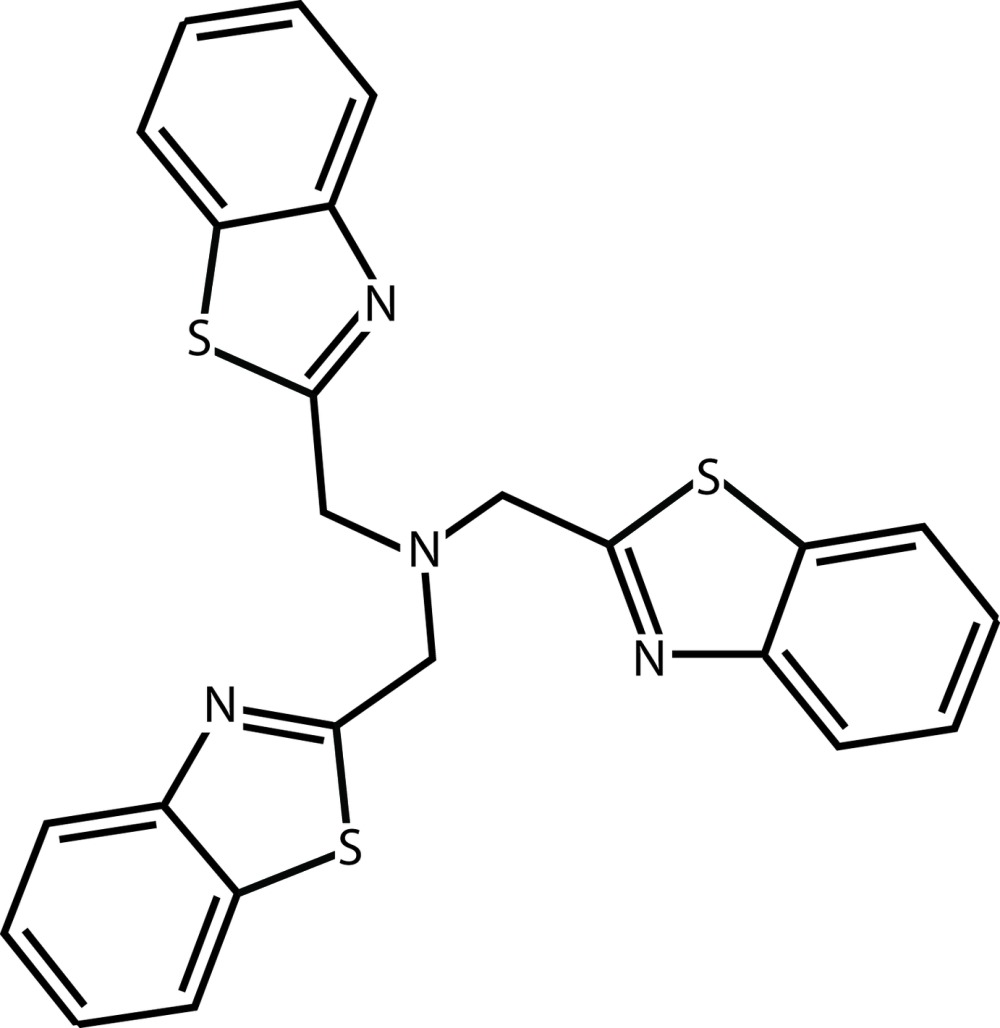


## Experimental   

### Crystal data  

C_24_H_18_N_4_S_3_
*M* *_r_* = 495.66
Triclinic, 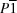

*a* = 6.6530 (3) Å
*b* = 14.3098 (6) Å
*c* = 14.5822 (7) Å
α = 61.471 (1)°
β = 88.474 (2)°
γ = 79.138 (1)°
*V* = 1194.61 (9) Å^3^
*Z* = 2
Mo *K*α radiation
μ = 0.34 mm^−1^
*T* = 100 K
0.15 × 0.12 × 0.10 mm

### Data collection  

Bruker APEXII CCD diffractometer
Absorption correction: multi-scan (*SADABS*; Bruker, 2005 ▸) *T*_min_ = 0.951, *T*_max_ = 0.967
4691 measured reflections
4691 independent reflections
3767 reflections with *I* > 2σ(*I*)
*R*_int_ = 0.000

### Refinement  

*R*[*F* ^2^ > 2σ(*F* ^2^)] = 0.038
*wR*(*F* ^2^) = 0.110
*S* = 1.08
4691 reflections
280 parameters
H-atom parameters constrained
Δρ_max_ = 0.28 e Å^−3^
Δρ_min_ = −0.29 e Å^−3^

### 

Data collection: *APEX2* (Bruker, 2005 ▸); cell refinement: *SAINT* (Bruker, 2005 ▸); data reduction: *SAINT*; program(s) used to solve structure: *SHELXS97* (Sheldrick, 2008 ▸); program(s) used to refine structure: *SHELXL97* (Sheldrick, 2008 ▸); molecular graphics: *SHELXTL* (Sheldrick, 2008 ▸); software used to prepare material for publication: *publCIF* (Westrip, 2010 ▸) and *PLATON* (Spek, 2009 ▸).

## Supplementary Material

Crystal structure: contains datablock(s) I, New_Global_Publ_Block. DOI: 10.1107/S2056989015017417/ff2141sup1.cif

Structure factors: contains datablock(s) I. DOI: 10.1107/S2056989015017417/ff2141Isup2.hkl

Supporting information file. DOI: 10.1107/S2056989015017417/ff2141Isup3.txt

Supporting information file. DOI: 10.1107/S2056989015017417/ff2141Isup4.cml

**Fig. 1.**
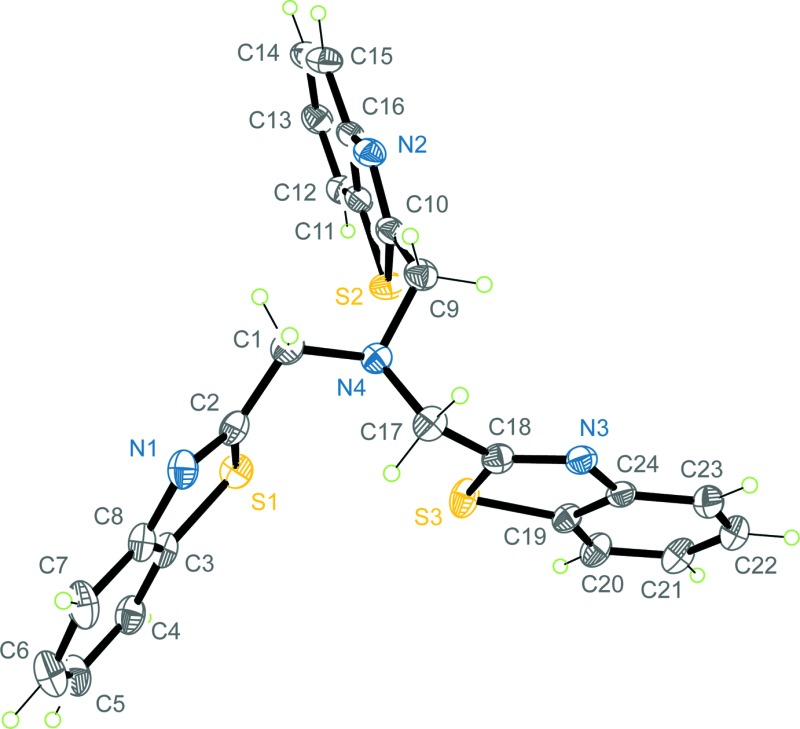
Molecular structure of the title compound, showing the atom labelling scheme. Displacement ellipsoids are drawn at the 50% probability level. H atoms are presented as spheres of arbitrary radius.

. DOI: 10.1107/S2056989015017417/ff2141fig1.tif
Mol­ecular structure of the title compound, showing the atom labelling scheme. Displacement ellipsoids are drawn at the 50% probability level. H atoms are presented as spheres of arbitrary radius.

**Fig. 2.**
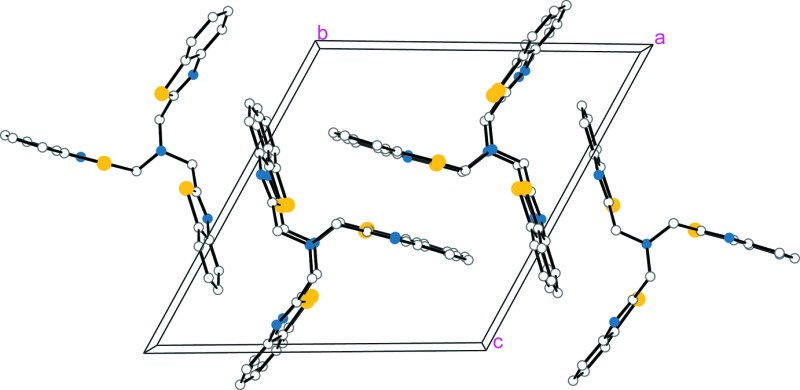
Molecular packing of the title compound.

. DOI: 10.1107/S2056989015017417/ff2141fig2.tif
Mol­ecular packing of the title compound.

CCDC reference: 1425576

Additional supporting information:  crystallographic information; 3D view; checkCIF report

## Figures and Tables

**Table 1:** Hydrogen-bond geometry (, )

*D*H*A*	*D*H	H*A*	*D* *A*	*D*H*A*
C4H4*A*N1^i^	0.95	2.47	3.376(3)	159
C12H12*A*N2^i^	0.95	2.60	3.449(2)	150
C20H20*A*N3^i^	0.95	2.54	3.490(3)	178

Symmetry code: (i) 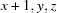.

## supplementary crystallographic information

### S1. Chemical context

Tripodal ligands with nitro­gen donors have become a common motif in coordination chemistry. Herein we report the structure of tris­(benzo­thia­zolyl­methyl)­amine. The bond distances and angles of the complex are similar to the previously reported bis­(benzo­thia­zol-2-yl­methyl)­amine (Zhang *et al.*, 2009). Copper and cobalt complexes of this ligand have been synthesized (Bautista & Thompson, 1980; Thompson *et al.*, 1980, Pandey & Mathur, 1995) and copper complexes have been explored as catalysts for azide-alkyne cyclo­additions (Rodionov, Presolski, Diaz, *et al.*, 2007; Rodionov, Presolski, Gardinier, *et al.* 2007).

The molecular structure of the title compound is shown in Figure 1. The compound possesses three planar benzo­thia­zoles that demonstrate a pseudo-*C_3_* configuration. The planes of the three benzo­thia­zole ligands exhibit dihedral angles of 112.555 (2), 123.744 (2) and 124.677 (3). The structure exhibits infinite chains along [100] which result from three C—H···N short contacts. The packing of the title compound is shown in Figure 2.

### S2. Synthesis and crystallization

The compound was prepared by literature procedure (Thompson *et al.*, 1980). Crystals suitable for single-crystal X-ray analysis were grown by slow evaporation of a di­ethyl ether solution.

### S3. Refinement details

The structure was solved by direct methods and all non-hydrogen atoms were refined anisotropically by full matrix least squares on F^2^. Hydrogen atoms were placed in calculated positions and then refined with riding models with C—H lengths of 0.99 Å for (CH_2_) and 0.95 Å for (CH) with isotropic displacement parameters set to 1.20 times *U*_eq_ of the parent C atoms. Diffused solvent (ethyl ether) was treated using Platon (Spek, 2009) program SQUEEZE (found void 157Å^3^, 48 electrons) and the unit card was adjusted by C_4_H_10_O to address issues of chemical formula, molecular mass, density and F000 value.

### Crystal data

**Table:**

C_24_H_18_N_4_S_3_	*Z* = 2
*M**_r_* = 495.66	*F*(000) = 518
Triclinic, *P*1	*D*_x_ = 1.378 Mg m^−^^3^
Hall symbol: -P 1	Mo *K*α radiation, λ = 0.71073 Å
*a* = 6.6530 (3) Å	Cell parameters from 6510 reflections
*b* = 14.3098 (6) Å	θ = 3.1–25.7°
*c* = 14.5822 (7) Å	µ = 0.34 mm^−^^1^
α = 61.471 (1)°	*T* = 100 K
β = 88.474 (2)°	Block, yellow
γ = 79.138 (1)°	0.15 × 0.12 × 0.10 mm
*V* = 1194.61 (9) Å^3^	

### Data collection

**Table:**

Bruker APEXII CCD diffractometer	4691 independent reflections
Radiation source: fine-focus ROTATING ANODE	3767 reflections with *I* > 2σ(*I*)
Graphite monochromator	*R*_int_ = 0.0000
φ and ω scans	θ_max_ = 26.0°, θ_min_ = 1.6°
Absorption correction: multi-scan (*SADABS*; Bruker, 2005)	*h* = −8→8
*T*_min_ = 0.951, *T*_max_ = 0.967	*k* = −15→17
4691 measured reflections	*l* = 0→17

### Refinement

**Table:**

Refinement on *F*^2^	Primary atom site location: structure-invariant direct methods
Least-squares matrix: full	Secondary atom site location: difference Fourier map
*R*[*F*^2^ > 2σ(*F*^2^)] = 0.038	Hydrogen site location: inferred from neighbouring sites
*wR*(*F*^2^) = 0.110	H-atom parameters constrained
*S* = 1.08	*w* = 1/[σ^2^(*F*_o_^2^) + (0.0612*P*)^2^ + 0.0926*P*] where *P* = (*F*_o_^2^ + 2*F*_c_^2^)/3
4691 reflections	(Δ/σ)_max_ < 0.001
280 parameters	Δρ_max_ = 0.28 e Å^−^^3^
0 restraints	Δρ_min_ = −0.29 e Å^−^^3^

### Special details

**Table:**

Geometry. All e.s.d.'s (except the e.s.d. in the dihedral angle between two l.s. planes) are estimated using the full covariance matrix. The cell e.s.d.'s are taken into account individually in the estimation of e.s.d.'s in distances, angles and torsion angles; correlations between e.s.d.'s in cell parameters are only used when they are defined by crystal symmetry. An approximate (isotropic) treatment of cell e.s.d.'s is used for estimating e.s.d.'s involving l.s. planes.
Refinement. Refinement of *F*^2^ against ALL reflections. The weighted *R*-factor *wR* and goodness of fit *S* are based on *F*^2^, conventional *R*-factors *R* are based on *F*, with *F* set to zero for negative *F*^2^. The threshold expression of *F*^2^ > σ(*F*^2^) is used only for calculating *R*-factors(gt) *etc*. and is not relevant to the choice of reflections for refinement. *R*-factors based on *F*^2^ are statistically about twice as large as those based on *F*, and *R*- factors based on ALL data will be even larger.

### Fractional atomic coordinates and isotropic or equivalent isotropic displacement parameters (Å^2^)

**Table:**

	*x*	*y*	*z*	*U*_iso_*/*U*_eq_	
S1	0.40279 (7)	0.15702 (4)	0.47208 (4)	0.04313 (15)	
S2	0.27604 (8)	0.38911 (4)	0.15881 (4)	0.04461 (16)	
S3	0.25502 (7)	0.44886 (4)	0.38876 (5)	0.04767 (16)	
N1	0.1195 (2)	0.05355 (12)	0.57200 (13)	0.0416 (4)	
N2	−0.0211 (2)	0.34050 (12)	0.08966 (12)	0.0405 (4)	
N3	−0.1239 (2)	0.54789 (12)	0.36542 (12)	0.0382 (4)	
N4	−0.0043 (2)	0.30973 (12)	0.34526 (12)	0.0368 (4)	
C1	0.0012 (3)	0.19336 (15)	0.39103 (15)	0.0418 (5)	
H1B	0.0358	0.1702	0.3375	0.050*	
H1A	−0.1361	0.1784	0.4139	0.050*	
C2	0.1562 (3)	0.13010 (14)	0.48240 (15)	0.0384 (4)	
C3	0.4652 (3)	0.05555 (14)	0.60010 (15)	0.0387 (4)	
C4	0.6507 (3)	0.01905 (15)	0.66060 (18)	0.0483 (5)	
H4A	0.7686	0.0480	0.6318	0.058*	
C5	0.6574 (3)	−0.06029 (16)	0.76342 (18)	0.0558 (6)	
H5A	0.7813	−0.0853	0.8064	0.067*	
C6	0.4877 (4)	−0.10437 (17)	0.8055 (2)	0.0628 (6)	
H6A	0.4962	−0.1581	0.8770	0.075*	
C7	0.3052 (3)	−0.07124 (16)	0.74462 (18)	0.0570 (6)	
H7A	0.1903	−0.1035	0.7731	0.068*	
C8	0.2940 (3)	0.00976 (13)	0.64160 (16)	0.0403 (4)	
C9	−0.1080 (3)	0.37025 (16)	0.23985 (15)	0.0424 (5)	
H9A	−0.1536	0.4475	0.2214	0.051*	
H9B	−0.2311	0.3412	0.2388	0.051*	
C10	0.0308 (3)	0.36203 (14)	0.16109 (14)	0.0374 (4)	
C11	0.3140 (3)	0.37193 (14)	0.04962 (14)	0.0375 (4)	
C12	0.4860 (3)	0.37765 (15)	−0.00699 (15)	0.0440 (5)	
H12A	0.6035	0.3968	0.0099	0.053*	
C13	0.4821 (3)	0.35475 (16)	−0.08855 (15)	0.0473 (5)	
H13A	0.5984	0.3582	−0.1284	0.057*	
C14	0.3113 (3)	0.32676 (17)	−0.11319 (16)	0.0521 (5)	
H14A	0.3129	0.3104	−0.1692	0.063*	
C15	0.1381 (3)	0.32219 (17)	−0.05775 (16)	0.0501 (5)	
H15A	0.0205	0.3039	−0.0758	0.060*	
C16	0.1393 (3)	0.34469 (14)	0.02442 (14)	0.0377 (4)	
C17	−0.0894 (3)	0.35415 (15)	0.41340 (16)	0.0411 (4)	
H17A	−0.0500	0.2993	0.4875	0.049*	
H17B	−0.2410	0.3719	0.4032	0.049*	
C18	−0.0096 (3)	0.45492 (15)	0.38820 (14)	0.0375 (4)	
C19	0.2093 (3)	0.58523 (15)	0.35501 (14)	0.0385 (4)	
C20	0.3495 (3)	0.65143 (16)	0.33751 (17)	0.0477 (5)	
H20A	0.4930	0.6233	0.3468	0.057*	
C21	0.2730 (4)	0.75976 (17)	0.30610 (18)	0.0531 (5)	
H21A	0.3657	0.8073	0.2922	0.064*	
C22	0.0642 (3)	0.80027 (16)	0.29458 (17)	0.0521 (5)	
H22A	0.0159	0.8752	0.2726	0.062*	
C23	−0.0763 (3)	0.73355 (16)	0.31451 (17)	0.0486 (5)	
H23A	−0.2196	0.7617	0.3073	0.058*	
C24	−0.0023 (3)	0.62444 (14)	0.34531 (14)	0.0364 (4)	

### Atomic displacement parameters (Å^2^)

**Table:**

	*U*^11^	*U*^22^	*U*^33^	*U*^12^	*U*^13^	*U*^23^
S1	0.0398 (3)	0.0435 (3)	0.0487 (3)	−0.0190 (2)	0.0104 (2)	−0.0209 (2)
S2	0.0367 (3)	0.0614 (3)	0.0469 (3)	−0.0167 (2)	0.0027 (2)	−0.0324 (3)
S3	0.0343 (3)	0.0435 (3)	0.0722 (4)	−0.0075 (2)	0.0131 (2)	−0.0339 (3)
N1	0.0365 (9)	0.0334 (8)	0.0527 (10)	−0.0113 (7)	0.0097 (8)	−0.0179 (8)
N2	0.0331 (8)	0.0457 (9)	0.0415 (9)	−0.0078 (7)	−0.0041 (7)	−0.0197 (8)
N3	0.0354 (8)	0.0461 (9)	0.0381 (9)	−0.0046 (7)	0.0009 (7)	−0.0253 (7)
N4	0.0352 (8)	0.0386 (8)	0.0390 (9)	−0.0090 (7)	0.0041 (7)	−0.0201 (7)
C1	0.0392 (11)	0.0432 (10)	0.0479 (12)	−0.0160 (8)	0.0061 (9)	−0.0232 (9)
C2	0.0373 (10)	0.0346 (9)	0.0504 (12)	−0.0123 (8)	0.0103 (9)	−0.0247 (9)
C3	0.0369 (10)	0.0308 (9)	0.0528 (12)	−0.0070 (8)	0.0085 (9)	−0.0237 (9)
C4	0.0384 (11)	0.0405 (10)	0.0672 (15)	−0.0057 (9)	0.0051 (10)	−0.0277 (11)
C5	0.0469 (13)	0.0415 (11)	0.0648 (15)	0.0036 (9)	−0.0063 (11)	−0.0183 (11)
C6	0.0592 (15)	0.0426 (12)	0.0606 (15)	0.0021 (11)	0.0007 (12)	−0.0085 (11)
C7	0.0500 (13)	0.0374 (11)	0.0658 (15)	−0.0093 (9)	0.0130 (11)	−0.0110 (11)
C8	0.0378 (10)	0.0288 (9)	0.0525 (12)	−0.0066 (8)	0.0088 (9)	−0.0184 (9)
C9	0.0331 (10)	0.0485 (11)	0.0446 (11)	−0.0055 (8)	−0.0005 (8)	−0.0225 (9)
C10	0.0324 (10)	0.0362 (9)	0.0390 (11)	−0.0056 (8)	−0.0043 (8)	−0.0146 (8)
C11	0.0368 (10)	0.0353 (9)	0.0334 (10)	−0.0069 (8)	−0.0041 (8)	−0.0109 (8)
C12	0.0406 (11)	0.0473 (11)	0.0415 (11)	−0.0137 (9)	0.0020 (9)	−0.0176 (9)
C13	0.0490 (12)	0.0500 (12)	0.0358 (11)	−0.0078 (9)	0.0037 (9)	−0.0158 (9)
C14	0.0560 (14)	0.0618 (13)	0.0373 (11)	−0.0071 (11)	−0.0024 (10)	−0.0244 (10)
C15	0.0468 (12)	0.0616 (13)	0.0448 (12)	−0.0143 (10)	−0.0070 (10)	−0.0263 (10)
C16	0.0376 (10)	0.0362 (9)	0.0334 (10)	−0.0043 (8)	−0.0069 (8)	−0.0126 (8)
C17	0.0345 (10)	0.0463 (11)	0.0481 (12)	−0.0117 (8)	0.0106 (9)	−0.0261 (9)
C18	0.0350 (10)	0.0444 (10)	0.0371 (10)	−0.0087 (8)	0.0070 (8)	−0.0228 (9)
C19	0.0393 (11)	0.0428 (10)	0.0401 (11)	−0.0071 (8)	0.0080 (8)	−0.0258 (9)
C20	0.0420 (11)	0.0521 (12)	0.0616 (13)	−0.0119 (9)	0.0134 (10)	−0.0368 (11)
C21	0.0573 (14)	0.0525 (12)	0.0647 (15)	−0.0193 (10)	0.0162 (11)	−0.0379 (11)
C22	0.0600 (14)	0.0419 (11)	0.0588 (14)	−0.0065 (10)	0.0019 (11)	−0.0290 (10)
C23	0.0453 (12)	0.0484 (11)	0.0535 (13)	0.0009 (9)	−0.0042 (10)	−0.0290 (10)
C24	0.0387 (10)	0.0426 (10)	0.0330 (10)	−0.0058 (8)	0.0010 (8)	−0.0230 (8)

### Geometric parameters (Å, º)

**Table:**

S1—C3	1.729 (2)	C7—H7A	0.9500
S1—C2	1.7415 (18)	C9—C10	1.489 (3)
S2—C11	1.729 (2)	C9—H9A	0.9900
S2—C10	1.7423 (18)	C9—H9B	0.9900
S3—C19	1.7349 (18)	C11—C12	1.385 (3)
S3—C18	1.7460 (18)	C11—C16	1.403 (3)
N1—C2	1.297 (2)	C12—C13	1.378 (3)
N1—C8	1.397 (2)	C12—H12A	0.9500
N2—C10	1.290 (2)	C13—C14	1.383 (3)
N2—C16	1.401 (2)	C13—H13A	0.9500
N3—C18	1.292 (2)	C14—C15	1.384 (3)
N3—C24	1.398 (2)	C14—H14A	0.9500
N4—C1	1.462 (2)	C15—C16	1.382 (3)
N4—C9	1.466 (2)	C15—H15A	0.9500
N4—C17	1.466 (2)	C17—C18	1.506 (2)
C1—C2	1.493 (3)	C17—H17A	0.9900
C1—H1B	0.9900	C17—H17B	0.9900
C1—H1A	0.9900	C19—C20	1.386 (3)
C3—C4	1.396 (3)	C19—C24	1.398 (3)
C3—C8	1.399 (3)	C20—C21	1.381 (3)
C4—C5	1.377 (3)	C20—H20A	0.9500
C4—H4A	0.9500	C21—C22	1.382 (3)
C5—C6	1.382 (3)	C21—H21A	0.9500
C5—H5A	0.9500	C22—C23	1.388 (3)
C6—C7	1.388 (3)	C22—H22A	0.9500
C6—H6A	0.9500	C23—C24	1.391 (3)
C7—C8	1.384 (3)	C23—H23A	0.9500
			
C3—S1—C2	89.03 (9)	C12—C11—C16	121.21 (18)
C11—S2—C10	89.09 (9)	C12—C11—S2	129.24 (15)
C19—S3—C18	89.02 (9)	C16—C11—S2	109.49 (14)
C2—N1—C8	110.35 (15)	C13—C12—C11	118.16 (19)
C10—N2—C16	110.28 (15)	C13—C12—H12A	120.9
C18—N3—C24	110.23 (15)	C11—C12—H12A	120.9
C1—N4—C9	111.51 (14)	C12—C13—C14	120.96 (19)
C1—N4—C17	112.81 (14)	C12—C13—H13A	119.5
C9—N4—C17	112.20 (14)	C14—C13—H13A	119.5
N4—C1—C2	110.82 (14)	C13—C14—C15	121.1 (2)
N4—C1—H1B	109.5	C13—C14—H14A	119.4
C2—C1—H1B	109.5	C15—C14—H14A	119.4
N4—C1—H1A	109.5	C16—C15—C14	118.71 (19)
C2—C1—H1A	109.5	C16—C15—H15A	120.6
H1B—C1—H1A	108.1	C14—C15—H15A	120.6
N1—C2—C1	123.72 (17)	C15—C16—N2	125.52 (18)
N1—C2—S1	116.22 (15)	C15—C16—C11	119.80 (18)
C1—C2—S1	120.06 (13)	N2—C16—C11	114.66 (16)
C4—C3—C8	120.91 (18)	N4—C17—C18	109.94 (14)
C4—C3—S1	129.40 (15)	N4—C17—H17A	109.7
C8—C3—S1	109.69 (14)	C18—C17—H17A	109.7
C5—C4—C3	117.91 (19)	N4—C17—H17B	109.7
C5—C4—H4A	121.0	C18—C17—H17B	109.7
C3—C4—H4A	121.0	H17A—C17—H17B	108.2
C4—C5—C6	121.4 (2)	N3—C18—C17	124.56 (16)
C4—C5—H5A	119.3	N3—C18—S3	116.31 (14)
C6—C5—H5A	119.3	C17—C18—S3	119.13 (14)
C5—C6—C7	120.9 (2)	C20—C19—C24	121.92 (17)
C5—C6—H6A	119.6	C20—C19—S3	128.84 (15)
C7—C6—H6A	119.6	C24—C19—S3	109.24 (13)
C8—C7—C6	118.6 (2)	C21—C20—C19	117.64 (19)
C8—C7—H7A	120.7	C21—C20—H20A	121.2
C6—C7—H7A	120.7	C19—C20—H20A	121.2
C7—C8—N1	125.12 (18)	C20—C21—C22	121.2 (2)
C7—C8—C3	120.18 (19)	C20—C21—H21A	119.4
N1—C8—C3	114.70 (17)	C22—C21—H21A	119.4
N4—C9—C10	111.13 (15)	C21—C22—C23	121.23 (19)
N4—C9—H9A	109.4	C21—C22—H22A	119.4
C10—C9—H9A	109.4	C23—C22—H22A	119.4
N4—C9—H9B	109.4	C22—C23—C24	118.42 (19)
C10—C9—H9B	109.4	C22—C23—H23A	120.8
H9A—C9—H9B	108.0	C24—C23—H23A	120.8
N2—C10—C9	124.19 (17)	C23—C24—N3	125.21 (17)
N2—C10—S2	116.46 (15)	C23—C24—C19	119.57 (17)
C9—C10—S2	119.28 (14)	N3—C24—C19	115.20 (16)
			
C9—N4—C1—C2	163.44 (15)	C11—C12—C13—C14	0.0 (3)
C17—N4—C1—C2	−69.23 (19)	C12—C13—C14—C15	0.8 (3)
C8—N1—C2—C1	−179.97 (16)	C13—C14—C15—C16	−0.9 (3)
C8—N1—C2—S1	0.3 (2)	C14—C15—C16—N2	−178.09 (17)
N4—C1—C2—N1	132.22 (18)	C14—C15—C16—C11	0.3 (3)
N4—C1—C2—S1	−48.1 (2)	C10—N2—C16—C15	177.63 (18)
C3—S1—C2—N1	0.28 (15)	C10—N2—C16—C11	−0.8 (2)
C3—S1—C2—C1	−179.47 (15)	C12—C11—C16—C15	0.5 (3)
C2—S1—C3—C4	179.89 (18)	S2—C11—C16—C15	−177.11 (15)
C2—S1—C3—C8	−0.75 (14)	C12—C11—C16—N2	179.05 (16)
C8—C3—C4—C5	−2.5 (3)	S2—C11—C16—N2	1.42 (19)
S1—C3—C4—C5	176.78 (16)	C1—N4—C17—C18	155.22 (15)
C3—C4—C5—C6	1.2 (3)	C9—N4—C17—C18	−77.82 (19)
C4—C5—C6—C7	1.1 (4)	C24—N3—C18—C17	−179.50 (16)
C5—C6—C7—C8	−2.2 (3)	C24—N3—C18—S3	0.4 (2)
C6—C7—C8—N1	−178.05 (19)	N4—C17—C18—N3	126.01 (19)
C6—C7—C8—C3	0.9 (3)	N4—C17—C18—S3	−53.8 (2)
C2—N1—C8—C7	178.12 (19)	C19—S3—C18—N3	0.05 (15)
C2—N1—C8—C3	−0.9 (2)	C19—S3—C18—C17	179.91 (15)
C4—C3—C8—C7	1.4 (3)	C18—S3—C19—C20	−179.60 (19)
S1—C3—C8—C7	−177.98 (16)	C18—S3—C19—C24	−0.43 (14)
C4—C3—C8—N1	−179.48 (16)	C24—C19—C20—C21	−2.3 (3)
S1—C3—C8—N1	1.1 (2)	S3—C19—C20—C21	176.77 (16)
C1—N4—C9—C10	−79.28 (18)	C19—C20—C21—C22	1.3 (3)
C17—N4—C9—C10	153.06 (15)	C20—C21—C22—C23	0.2 (3)
C16—N2—C10—C9	176.60 (16)	C21—C22—C23—C24	−0.8 (3)
C16—N2—C10—S2	−0.2 (2)	C22—C23—C24—N3	−178.19 (18)
N4—C9—C10—N2	133.35 (18)	C22—C23—C24—C19	−0.2 (3)
N4—C9—C10—S2	−49.92 (19)	C18—N3—C24—C23	177.37 (18)
C11—S2—C10—N2	0.87 (15)	C18—N3—C24—C19	−0.7 (2)
C11—S2—C10—C9	−176.10 (15)	C20—C19—C24—C23	1.8 (3)
C10—S2—C11—C12	−178.60 (18)	S3—C19—C24—C23	−177.46 (14)
C10—S2—C11—C16	−1.23 (13)	C20—C19—C24—N3	179.98 (17)
C16—C11—C12—C13	−0.6 (3)	S3—C19—C24—N3	0.7 (2)
S2—C11—C12—C13	176.48 (14)		

### Hydrogen-bond geometry (Å, º)

**Table:**

*D*—H···*A*	*D*—H	H···*A*	*D*···*A*	*D*—H···*A*
C4—H4*A*···N1^i^	0.95	2.47	3.376 (3)	159
C12—H12*A*···N2^i^	0.95	2.60	3.449 (2)	150
C20—H20*A*···N3^i^	0.95	2.54	3.490 (3)	178

Symmetry code: (i) *x*+1, *y*, *z*.
