# Supplementary figures and images for: Crystal structure of N,N,N-tris­[(1,3-benzo­thia­zol-2-yl)meth­yl]amine
Source: Acta Crystallogr E Crystallogr Commun. 2015 Sep 26;71(Pt 10):o786–7. doi: 10.1107/S2056989015017417 (PMC4647366; doi:10.1107/S2056989015017417)

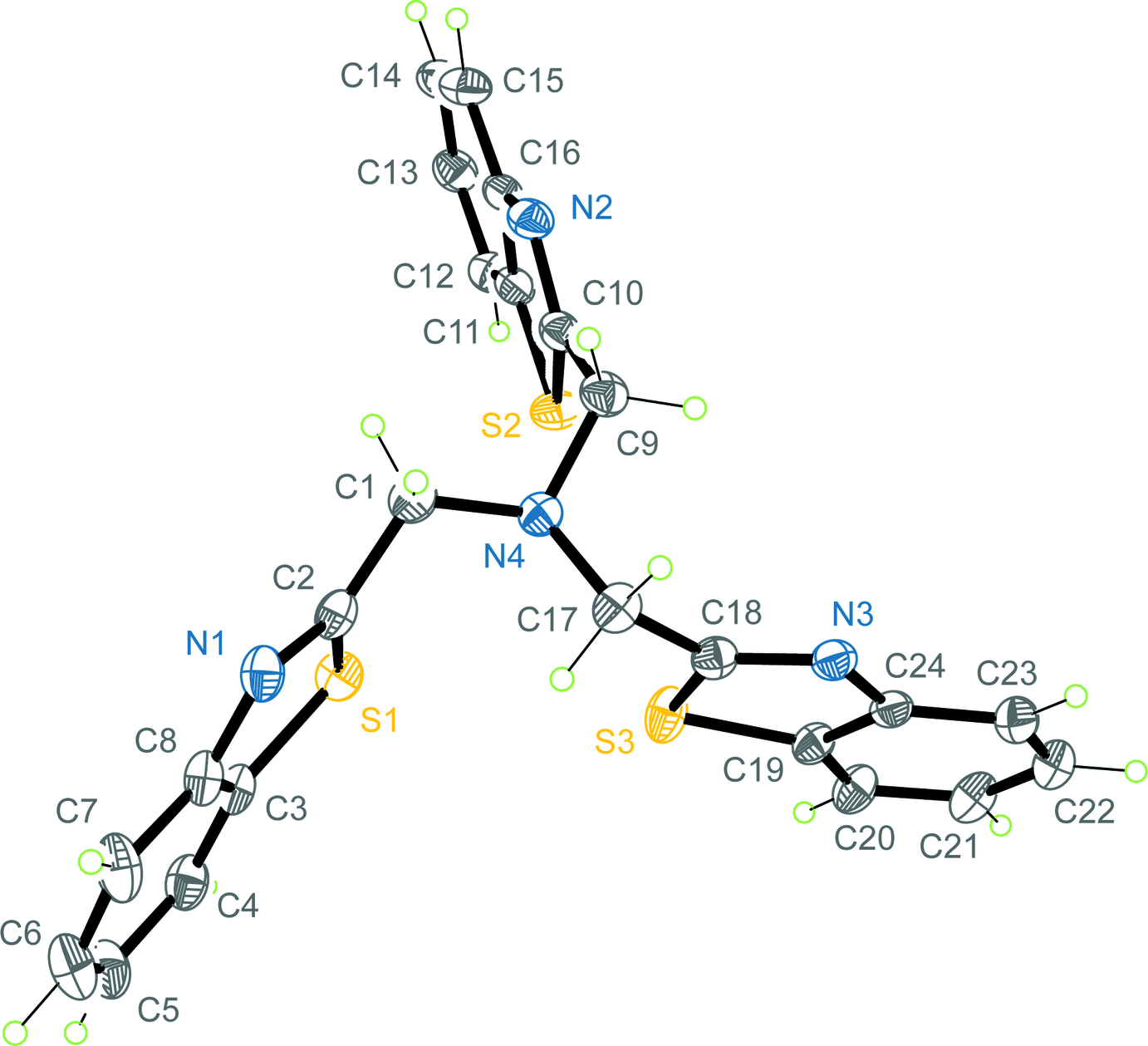

Supplement: Supplementary file 5 [file e-71-0o786-fig1.tif]

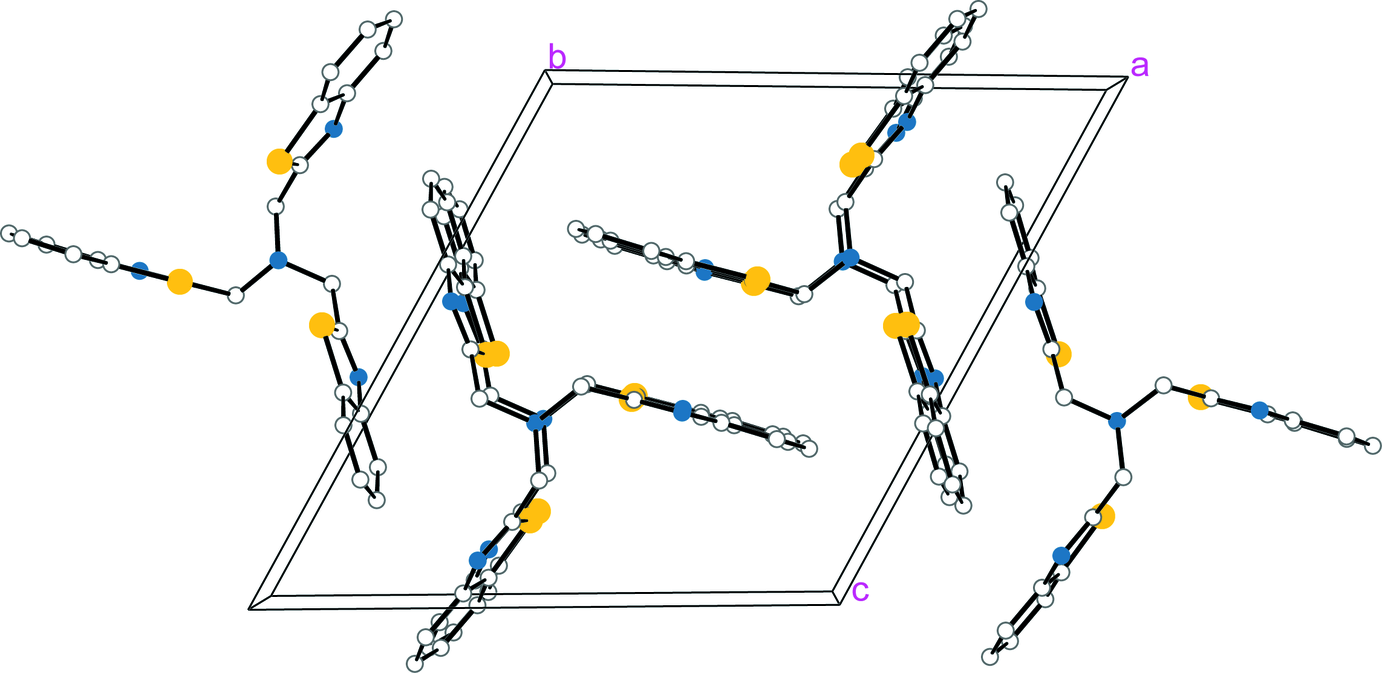

Supplement: Supplementary file 6 [file e-71-0o786-fig2.tif]
